# Supplementary figures and images for: A holistic evolutionary and structural study of flaviviridae provides insights into the function and inhibition of HCV helicase
Source: PeerJ. 2013 May 7;1:e74. doi: 10.7717/peerj.74 (PMC3646357; doi:10.7717/peerj.74)

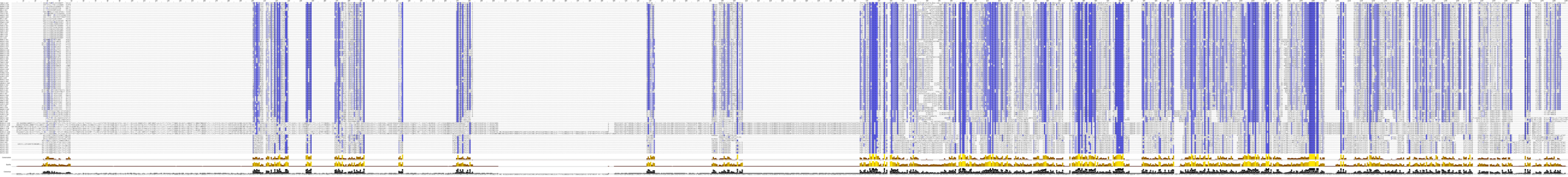

Supplement: Figure S1 — The alignment was generated using Muscle, and visualized with Jalview. Conserved residues are highlighted based on percent identity in the alignment (blue: >80% agreement, mid blue: >60% agreement, light blue: >40% agreement; only the residues that agree with the consensus residue for each column are coloured). [file peerj-01-74-s001.png]

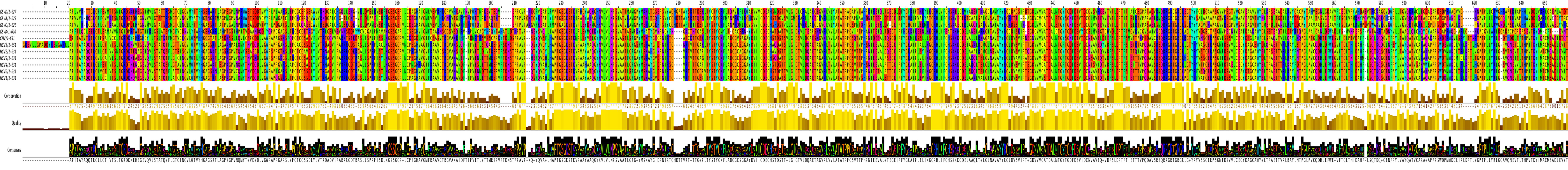

Supplement: Figure S2 — The alignment was generated using Muscle, and visualized with Jalview. Amino acids are colored blue based on the Taylor color scheme (i.e. acidic/basic/polar). [file peerj-01-74-s002.png]
