# Supplementary material for: A holistic evolutionary and structural study of flaviviridae provides insights into the function and inhibition of HCV helicase
Source: PeerJ. 2013 May 7;1:e74. doi: 10.7717/peerj.74 (PMC3646357; doi:10.7717/peerj.74)
Supplement: File S1 [file peerj-01-74-s003.docx]

>1A1V:A|PDBID|CHAIN|SEQUENCE

MVDFIPVENLETTMRSPVFTDNSSPPAVPQSFQVAHLHAPTGSGKSTKVPAAYAAQGYKVLVLNPSVAATLGFGAYMSKAHGVDPNIRTGVRTITTGSPITYSTYGKFLADGGCSGGAYDIIICDECHSTDATSILGIGTVLDQAETAGARLVVLATATPGSVTVPHPNIEEVALSTTGEIPFYGKAIPLEVIKGGRHLIFCHSKKKCDELAAKLVALGINAVAYYRGLDVSVIPTSGDVVVVATDALMTGFTGDFDSVIDCNTCVTQTVDFSLDPTFTIETTTLPQDAVSRTQRRGRTGRGKPGIYRFVAPGERPSGMFDSSVLCECYDAGCAWYELTPAETTVRLRAYMNTPGLPVCQDHLEFWEGVFTGLTHIDAHFLSQTKQSGENFPYLVAYQATVCARAQAPPPSWDQMWKCLIRLKPTLHGPTPLLYRLGAVQNEVTLTHPITKYIMTCMSADLEVVTGSGSHHHHHH
